# Lymphoma Spectrum of Image Findings in One Patient

**DOI:** 10.5334/jbsr.2979

**Published:** 2023-05-31

**Authors:** Kuan-Yu Lin, Wilson Tiu Lao, Wing P. Chan

**Affiliations:** 1 Wan Fang Hospital, Taipei Medical University, TW

**Keywords:** lymphoma, Hodgkin’s lymphoma, metastasis, neoplasm, cancer

## Abstract

Lymphoma is a malignancy arising from lymphocytes or lymphoblasts. It affects the lymphoid system and may be expressed in a variety of ways and behave in different fashions. Depending on the organ involved, aggressiveness, and primary or secondary disease, the expression of lymphoma shows polymorphism and sometimes makes it difficult to diagnose from imaging. This article will describe the image findings of lymphoma in different organ systems of one patient.

## Introduction

Lymphoma is a cancer that develops in the white blood cells of the lymphatic system. It can be restricted to the lymphatic system or arise as extranodal disease. The diagnosis of lymphoma is based on pathological histology and is distinguished on a morphological basis between classical Hodgkin’s lymphoma (HL), characterized by the presence of Reed-Sternberg cells, and non-Hodgkin’s lymphomas (NHL) [1]. Non-Hodgkin’s lymphomas account for 90% of all lymphomas, while HL constitutes the remaining 10% [2].

Hodgkin’s lymphoma is usually entirely confined to the lymph nodes and typically starts in the upper body. Extranodal HL, although uncommon, may be found in any organ system, either as a primary manifestation or as dissemination of systemic disease.

In addition to lymphoma, numerous conditions, such as other malignancy with metastasis, infectious diseases, and autoimmune disorders, can also cause lymphadenopathy and present similar image findings to lymphoma. Since HL is considered a curable malignancy and the differential diagnosis is broad, it is clinically important to discriminate HL from other conditions for patient management. In this article, the image findings in different organ systems of Hodgkin’s lymphoma in a patient will be described.

## Lymphoma Spectrum of Image Findings in One Patient

**Figure 1:**
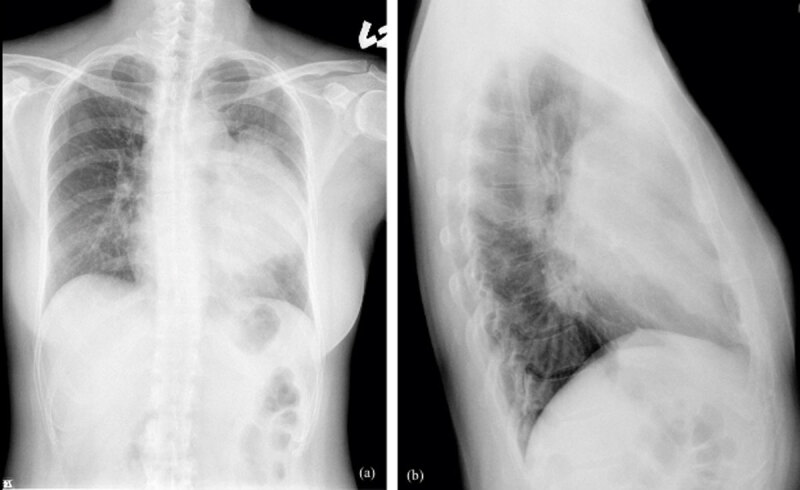
Chest radiographs PA **(a)** and lateral **(b)** views show a large anterior mediastinal mass lesion.

**Figure 2:**
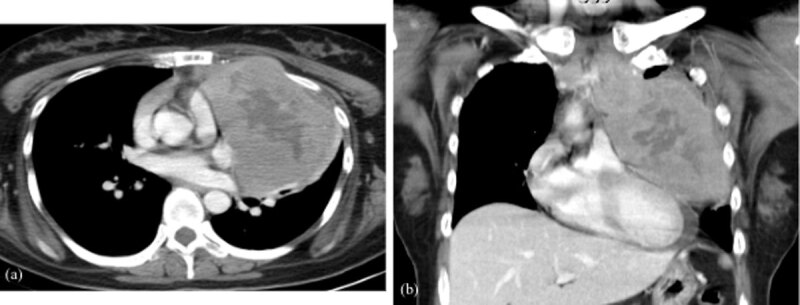
Post-contrast chest CT scan axial **(a)** and coronal **(b)** views: A large heterogeneous enhancing anterior mediastinal mass with intrinsic necrosis.

**Figure 3:**
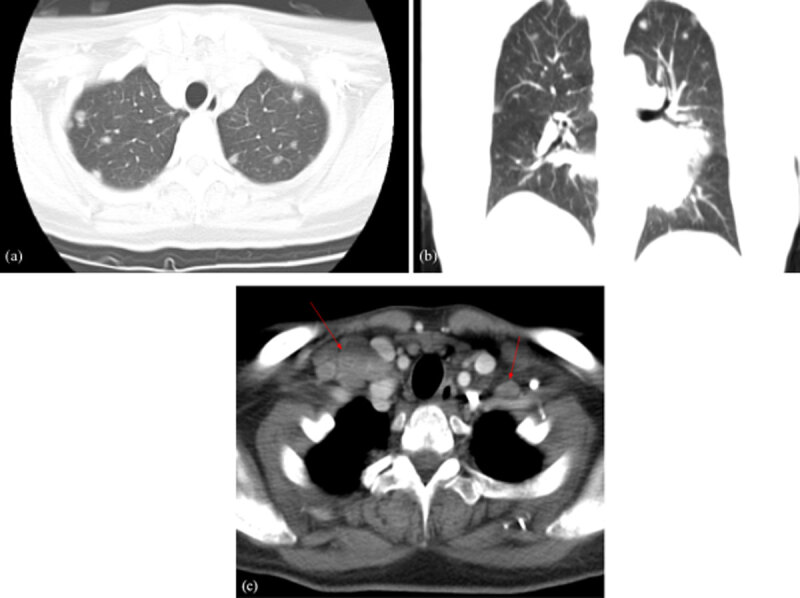
Chest CT scan, lung window, axial **(a)** and coronal **(b)** views: Multiple ground glass nodules at the bilateral lungs with peribronchovascular and subpleural distribution. Post-contrast chest CT scan **(c)**: Enlarged lymph nodes at bilateral clavicular fossa (arrows).

**Figure 4:**
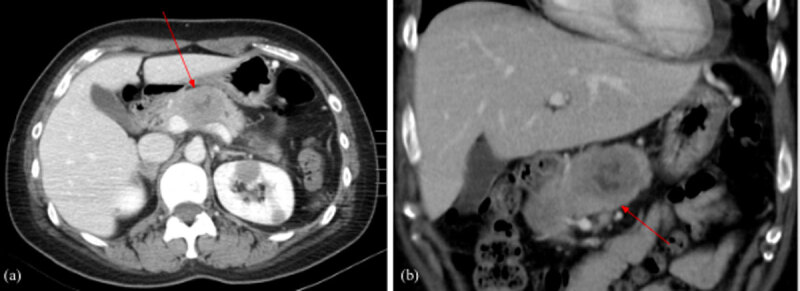
Contrast-enhanced abdominal CT scan axial **(a)** and coronal **(b)** views: Poor enhancing mass lesion at the body of pancreas with encasement of the superior mesenteric vein.

**Figure 5:**
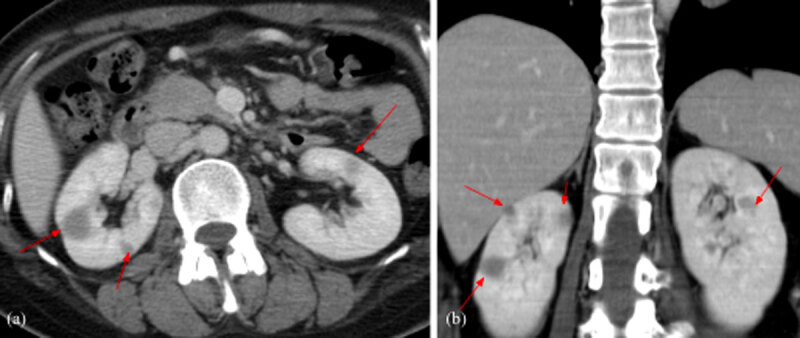
Contrast-enhanced abdominal CT scan axial **(a)** and coronal **(b)** views of the kidneys: Multiple poor enhancing low attenuation lesions in the bilateral kidneys.

A 31-year-old woman initially presented in the emergency room for left upper chest tightness. Her vital signs, blood exam, and electrocardiogram (EKG) were normal while chest radiographs revealed a large anterior mediastinal mass lesion (Figure 1). On chest and abdominal computed tomography (CT), a large heterogeneous anterior mediastinal mass with intrinsic necrosis and indistinct tissue planes was shown (Figure 2). Multiple small nodules with indistinct outline at the bilateral lungs; lymphadenopathy at bilateral supraclavicular, paratracheal, and aortopulmonary window regions; and a small amount of pericardial effusion were also noted (Figure 3). In addition, diffuse poor enhancing infiltrating mass lesion at the pancreas which encased the superior mesenteric vein was noted, but this did not cause occlusion nor dilatation of pancreatic duct or biliary tree (Figure 4). Multiple low-attenuation lesions in the bilateral kidneys were found (Figure 5).

Excisional biopsy of right supraclavicular enlarged lymph node was performed, and the pathologic result turned out classical Hodgkin’s lymphoma, mixed cellularity subtype.

**Figure 6:**
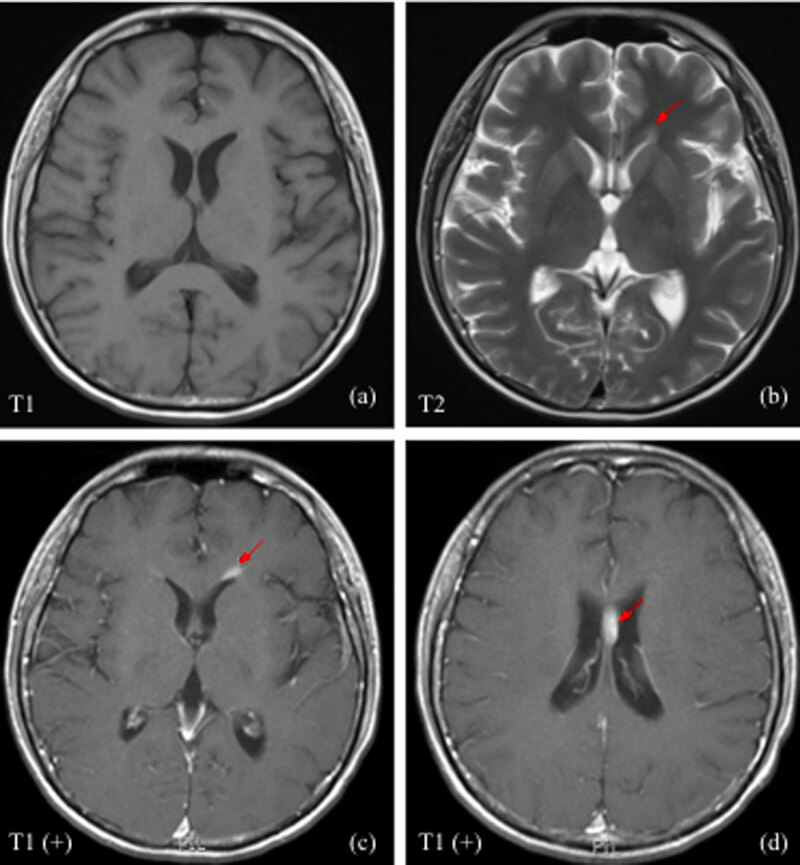
Contrast-enhanced brain MRI: Homogeneous enhancing lesions in the left periventricular region (arrow in c) and septum pellucidum (arrow in d) with isointense on T1WI **(a)** and hyperintense on T2WI **(b)**.

**Figure 7:**
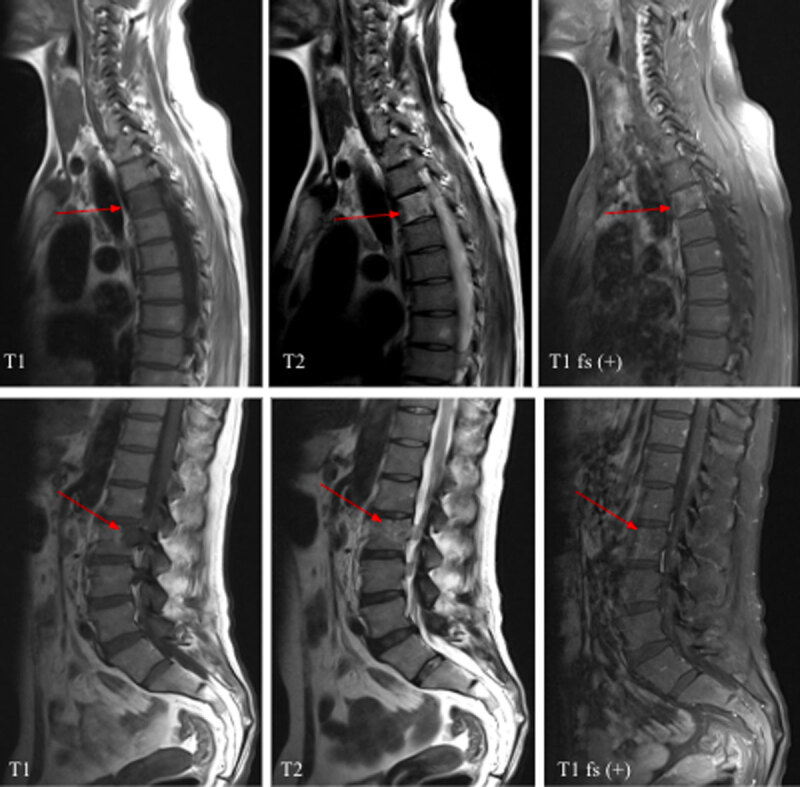
Pre- and post-contrast MRI: Enhancing lesions with hypointense on T1WI and iso/hyperintense on T2WI were noted at T3 and L2 vertebral bodies (arrows).

**Figure 8:**
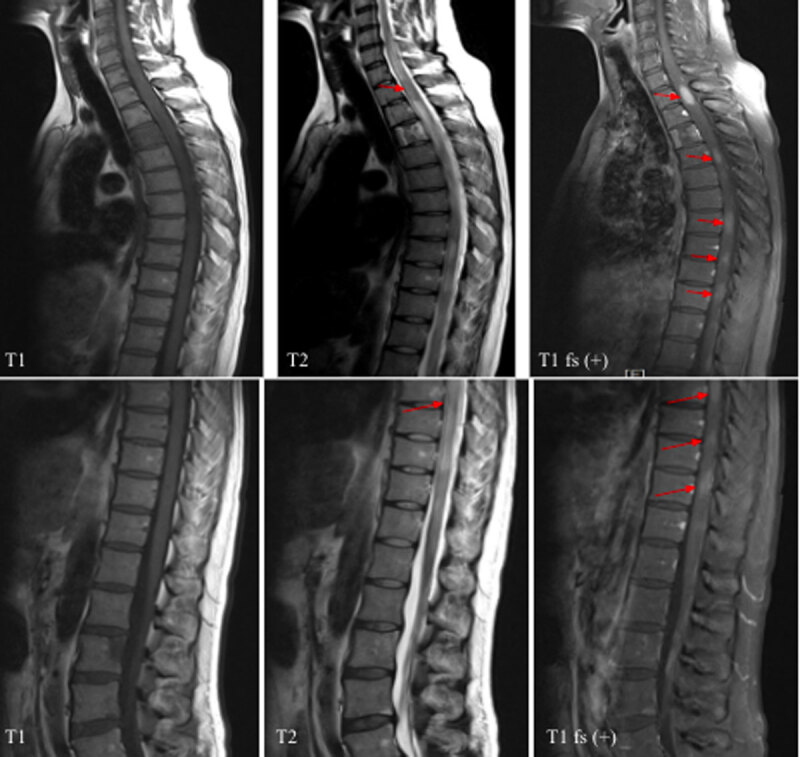
Pre- and post-contrast MRI: Multiple enhancing spinal cord lesions with isointense on T1WI and iso/hyperintense on T2WI (arrows).

For progressive back pain, bilateral lower-leg weakness, and urine retention noted during follow-up, enhanced magnetic resonance imaging (MRI) of the whole spine and the brain was performed. Enhanced brain MRI discovered homogeneous enhancing lesions in the left periventricular region and septum pellucidum, which had an isointense signal on T1 weighted image (T1WI) and hyperintense on T2 weighted image (T2WI) (Figure 6). The enhanced MRI of the whole spine revealed several enhancing lesions with hypointense signal on T1WI and iso/hyperintense on T2WI, involving the vertebral bodies, lamina, and pedicles of T3 and L2. Multiple abnormal enhancing spinal cord lesions with isointense signal on T1WI and iso-/hyperintense with surrounding edema on T2WI were also noted (Figures 7 and 8). These features in the spine and brain were compatible with lymphoma.

There are other conditions that can mimic the image findings in different organ systems in lymphoma, such as other malignancy, infection, and metastases. However, some image findings in lymphoma have distinct characteristics and may provide some clues for correct diagnosis. For example, lymphoma in mediastinum usually demonstrates a soft tissue attenuating mass, with smooth or lobulated margins which conform to surrounding structures and cystic areas on CT; pancreatic lymphoma can appear as a well-circumscribed discrete mass or as infiltrative appearance with poor enhancement, and it rarely causes obstruction or dilatation of the pancreatic duct or surrounding blood vessels [3].

## Conclusion

Differentiating between lymphoma and other similar-appearing diseases is paramount for the correct management of patients. Despite the fact that multiple lesions in different organ systems make it a challenge to distinguish lymphoma from other primary cancers, infection, or metastasis, there are a number of common imaging characteristics bound to suggest the diagnosis.
